# Cardiovascular disease in women with breast cancer – a nationwide cohort study

**DOI:** 10.1186/s12885-021-08716-5

**Published:** 2021-09-18

**Authors:** Marie Jakobsen, Christophe Kolodziejczyk, Morten Sall Jensen, Peter Bo Poulsen, Humma Khan, Thomas Kümler, Michael Andersson

**Affiliations:** 1 VIVE, The Danish Center for Social Science Research, Herluf Trolles Gade 11, DK-1152 Copenhagen K, Denmark; 2 VIVE, The Danish Center for Social Science Research, Oluf Palmes Allé 22, DK-8200 Aarhus N, Denmark; 3 Pfizer Denmark, Lautrupvang 8, DK-2750 Ballerup, Denmark; 4 Department of Cardiology, Herlev-Gentofte University Hospital, Borgmester Ib Juuls Vej 1, DK-2730 Herlev, Denmark; 5 Department of Oncology, Copenhagen University Hospital, Rigshospitalet, Blegdamsvej 9, DK-2100 Copenhagen Oe, Denmark

**Keywords:** Breast cancer, Cardiovascular disease, Prevalence, Incidence, Cohort study, Matched control group

## Abstract

**Background:**

There is increasing concern about cardiovascular disease (CVD) after breast cancer (BC). The aim of this study was to estimate the prevalence of different types of CVD in women diagnosed with BC compared to cancer-free controls as well as the incidence of CVD after BC diagnosis*.*

**Methods:**

We performed a cohort study based on data from national registries covering the entire Danish population. We followed 16,505 cancer-naïve BC patients diagnosed from 2003 to 2007 5 years before and up to 10 years after BC diagnosis compared to 165,042 cancer-free controls.

**Results:**

We found that 15.6% of BC patients were registered with at least one CVD diagnosis in hospital records before BC diagnosis. Overall, BC patients and controls were similar with regard to CVD comorbidity before BC diagnosis. After BC diagnosis, the incidence of all CVD diagnoses combined was significantly higher in BC patients than controls up to approximately 6 years after the index date (BC diagnosis). After 10 years, 28% of both BC patients and controls (without any CVD diagnosis up to 5 years before the index date) had at least one CVD diagnosis according to hospital records. However, the incidence of heart failure, thrombophlebitis/thrombosis and pulmonary heart disease including pulmonary embolism remained higher in BC patients than controls during the entire 10-year follow-up period. After 10 years, 2.7% of BC patients compared to 2.5% of controls were diagnosed with heart failure, 2.7% of BC patients compared to 1.5% of controls were diagnosed with thrombophlebitis/thrombosis, and 1.5% of BC patients compared to 1.0% of controls were diagnosed with pulmonary heart disease according to hospital records. Furthermore, we found that the risk of heart failure and thrombophlebitis/thrombosis was higher after chemotherapy.

**Conclusions:**

Focus on CVD in BC patients is important to ensure optimum treatment with regard to BC as well as possible CVD. Strategies to minimise and manage the increased risk of heart failure, thrombophlebitis/thrombosis and pulmonary heart disease including pulmonary embolism in BC patients are especially important.

**Supplementary Information:**

The online version contains supplementary material available at 10.1186/s12885-021-08716-5.

## Background

Breast cancer (BC) is the most prevalent type of cancer among women worldwide (https://gco.iarc.fr/today/data/factsheets/populations/900-world-fact-sheets.pdf). In Denmark, approximately 4700 women are diagnosed with BC every year, corresponding to 25% of all new primary cancer cases among women, and 10% of all women are diagnosed with BC before they become 75 years old (http://www-dep.iarc.fr/NORDCAN/DK/frame.asp).

Many patients have coexisting diseases (comorbidity) when they are diagnosed with cancer, especially elderly cancer patients [1]. International studies show that 20–35% of BC patients have one or more comorbidities at the time of BC diagnosis [2]. In Danish women diagnosed with early stage BC, the percentage with comorbidity has been shown to be approximately 20% [3, 4].

Most existing studies assess comorbidity in BC patients using an aggregated measure such as the Charlson Comorbidity Index (CCI) [2–7]. Fewer studies provide information on the prevalence and incidence of specific comorbid conditions and different types of CVD in particular [8–10]. Such information is critical to inform clinicians and ensure optimum treatment of BC patients with regard to BC as well as possible CVD.

Abdel-Qadir et al. [9] have shown that women diagnosed with early stage BC are more likely to have a history of heart failure, ischemic heart disease, cerebrovascular disease, peripheral vascular disease, atrial fibrillation and hypertension compared to cancer-free controls, and that the incidence of heart failure, cerebrovascular disease and arrhythmias is significantly higher in BC patients up to 10 years after BC diagnosis. Strongman et al. [10] find that the incidence of heart failure, venous thromboembolism and pericarditis is significantly higher in BC survivors compared to cancer-free controls up to 2 years after BC diagnosis, but do not find a higher incidence of stroke or arrhythmias as in the study by Abdel-Qadir et al. Both studies are based on registry data covering a segment of the population in Canada and the UK, respectively. Even though the segment of the UK population (6.9%) in the study by Strongman et al. has been shown to be broadly representative of the general UK population in terms of age, gender and ethnicity [11], a risk of selection bias remains as the study population can differ from the general population in other ways.

The overall aim of our study was to estimate the prevalence and incidence of different types of CVD in BC patients compared to cancer-free controls up to 5 years before and 10 years after BC diagnosis based on registry data covering the entire Danish population. Furthermore, we investigate the risk of developing CVD in BC patients following chemotherapy, radiation therapy, antibody therapy and hormonal therapy. Compared with previous studies, our study includes a larger and less segmented study population of BC patients and a more precisely matched control group.

## Methods

We performed a cohort study based on data from national registries covering the entire Danish population (5.8 million inhabitants). Data from the Danish Cancer Registry (CAR) [12], the Danish National Patient Registry (NPR) [13, 14], the Danish National Prescription Registry for Drugs (NPRD) [15, 16], Danish education registers [17] and the Danish Civil Registration System [18] were linked using the unique personal identification number assigned to every citizen in Denmark (known as the CPR number).

### Study population

We used CAR to identify all women who were 18+ years old and diagnosed with BC in Denmark during the period from 1 January 2003 to 31 December 2007. CAR is a research registry containing information at the individual level on the incidence of cancer in the Danish population since 1943 [12]. Diagnoses in CAR are coded according to the International Classification of Diseases 7th Revision (ICD-7) from 1943 to 1978 and according to the International Classification of Diseases 10th Revision (ICD-10) from 1978 and onwards [12]. We used the diagnosis code C50* (ICD-10) to identify BC patients in the study population. Women who were registered in CAR with any cancer diagnosis (ICD-10: C00*-C97* or ICD-7: 140*-207*) prior to the BC diagnosis or who had lived outside Denmark for more than 1 year when they were 18+ years old were excluded to allow focus on newly diagnosed BC patients who had no previous cancer diagnosis (cancer-naïve BC patients). The index date was that of BC diagnosis in CAR.

Subgroups of BC patients were defined according to age group (< 45, 45–74 and 75+) and cancer disease stage (stage I-III, stage IV or unknown) at index date. Data on cancer disease stage at index date were obtained from CAR.

Exact matching was used to identify a control group of women from the general population in Denmark without any cancer diagnosis in CAR (ICD-10: C00*-C97* or ICD-7: 140*-207*) up to and including the index year (i.e. the year of BC diagnosis of the matched BC patient). For each BC patient, we identified 10 controls matched according to age group (< 30, 30–34, 35–39, 40–44, 45–49, 50–54, 55–59, 60–64, 65–69, 70–74, 75–80, 80–85, 85+), region of residence (Capital Region, Region Zealand, Region of Southern Denmark, Central Jutland Region, North Jutland Region) and education (low, medium, high, unknown). Each person in the control group was assigned a random index date during the index year.

We followed BC patients and controls up to10 years from the index date. BC patients and controls who died during the 10-year follow-up period were censored at time of death. There was no loss to follow-up as national registry data covering the whole Danish population were used to assess status at follow-up.

### Outcomes and covariates

**Fig. 1:**
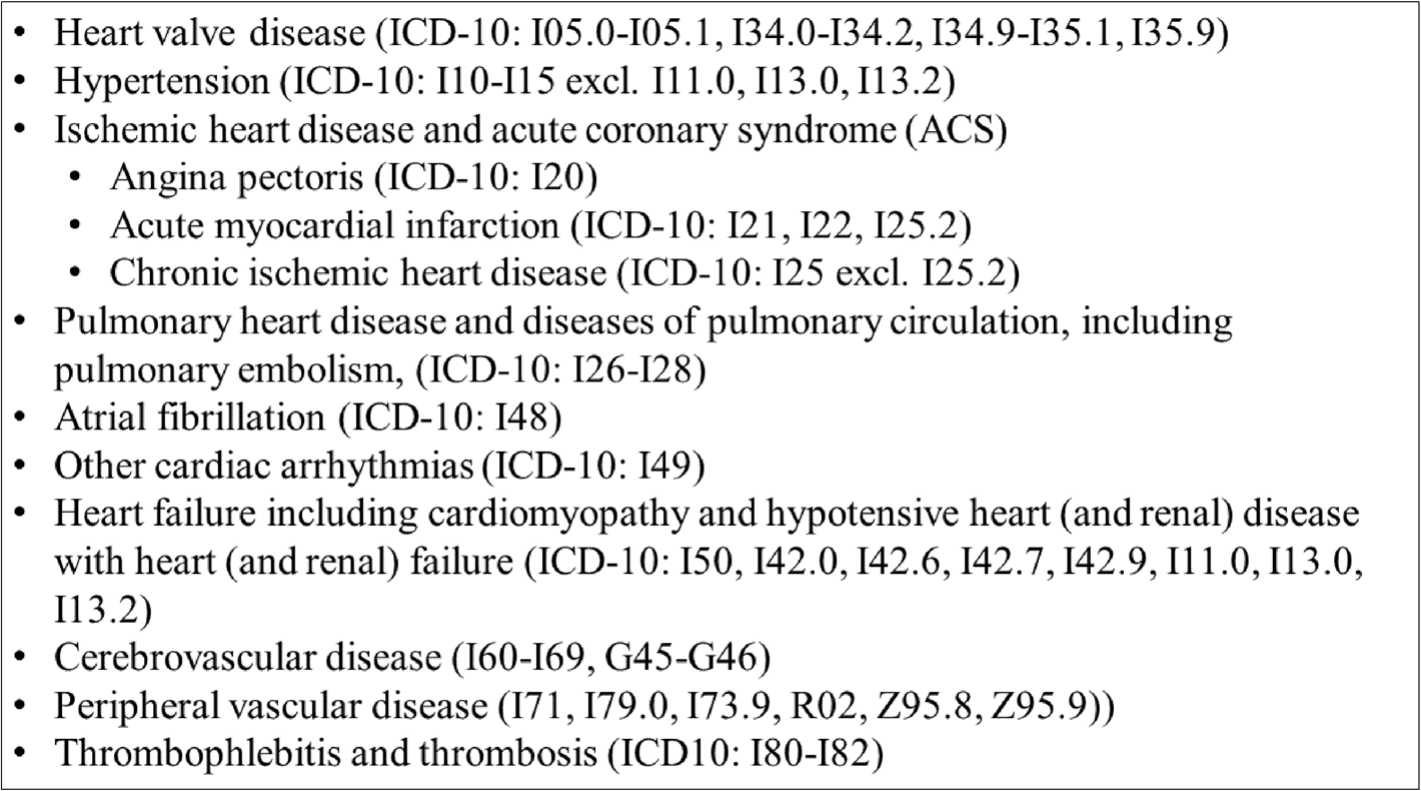
Types of CVD included in the study (ICD-10 diagnoses)

We defined CVD as at least one primary or secondary CVD diagnosis in NPR, see Fig. 1. NPR holds information on all patients discharged from Danish hospitals since 1977 and on emergency department and outpatient visits since 1995 [14]. For each hospital contact, one primary and optional secondary diagnoses are registered according to the International Classification of Diseases (ICD). The primary diagnosis is the main reason for the hospital contact and secondary diagnoses identify other relevant diseases.

**Fig. 2:**
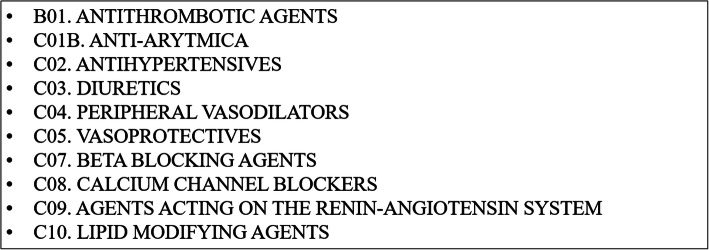
CVD prescription drugs included in the study (ATC groups)

Furthermore, we identified BC patients with at least two CVD drug prescriptions (i.e. drugs related to CVD disease management) within the same year in NPRD, see Fig. 2. NPRD includes information on all primary care prescription drugs dispensed from community pharmacies in Denmark coded according to the Anatomical Therapeutic Chemical Classification System (ATC) [16]. We applied the criteria of two prescriptions to exclude persons who were apparently not in continuous treatment. It is not straightforward to identify CVD patients based on NPRD data as the drugs listed in Box 2 are not used exclusively for CVD disease management, and the NPRD does not contain the same information on diagnoses as NPR. However, NPRD data are still relevant to capture the large number of CVD patients who are not registered in NPR because their condition does not require hospitalization or outpatient visits to hospitals.

We used CCI as an aggregate measure of overall comorbidity [19] and diabetes at index date as covariates in multivariate Cox regression analyses. We calculated CCI using information from NPR on primary and secondary diagnoses up to 5 years before the index date. We classified the study population into three groups with a CCI score equal to zero, 1, or 2 and above, respectively. We defined diabetes at index date as at least one primary or secondary diabetes diagnosis in NPR (ICD-10: E10*-E11*) up to 5 years prior to the index date or at least two insulin prescriptions or prescriptions for blood glucose lowering drugs in NPRD (ATC group A10A* and A10B*) within the same year during the 5-year period.

We identified persons in the BC group who had received different types of cancer treatments based on data from NPR. Surgery was defined by a ‘surgery procedure code’ in combination with BC diagnosis. Chemotherapy was defined by a BWHA procedure code, radiation therapy was defined by a BWG procedure code, antibody therapy was defined by a BOHJI procedure code, and hormonal therapy was defined by a BWHC procedure code.

We identified persons in the BC and control group who died during follow-up (including date of death) based on data from the Civil Registration System.

Data on age, region of residence and education were obtained from the Civil Registration System and education registers.

### Statistical analysis

CVD prevalence rates were calculated as number of persons with at least one CVD primary or secondary diagnosis in NPR up to 5 years prior to the index date per 1000 persons for all CVD diagnoses combined and specific types of CVD. Furthermore, number of BC patients and cancer-free controls treated with CVD prescription drugs up to 5 years prior to the index date per 1000 persons were calculated based on NPRD data.

The cumulative incidence of all CVD diagnoses combined as well as specific types of CVD were calculated up to 10 years after the index date (BC diagnosis) allowing for the presence of competing risk of death [20]*.* A competing risk is an event whose occurrence precludes another event of interest. CVD incidence rates for BC patients and controls were calculated among BC patients and controls without CVD diagnosis up to 5 years before the index date.

We performed multivariate Cox regression analyses to investigate the risk of developing CVD in BC patients following chemotherapy, radiation therapy, antibody therapy and hormonal therapy initiated within 9 months after BC diagnosis. The risk of CVD was calculated based on either CVD diagnoses in NPR (model 1) or CVD prescription drugs in NPDR (model 2). Only BC patients without any CVD diagnosis in NPR 5 years prior to the index date were included in model 1, and only BC patients without a history of CVD prescription drugs 5 years prior to the index date were included in model 2. We included time dependent exposure dummies that classified the patients’ time-to-event’ as unexposed until treatment began to avoid immortal time bias [21]. BC patients who had received surgery, but not the cancer treatment in question (chemotherapy, radiation therapy, antibody therapy or hormonal therapy) were used as reference. Adjustments were made for differences between groups with regard to age, region of residence, education and cohort (year of BC diagnosis) as well as comorbidity. Persons who died during follow-up were censored at time of death.

The analyses were performed using Stata 14 (StataCorp, College Station, TX). The statistical significance of differences between groups were evaluated using two-sided t-tests with a 5% level of significance or 95% confidence intervals.

There were no missing data except for education. Statistical analyses were carried out using a special category for this group (education unknown).

## Results

The study population included 16,505 BC patients and 165,042 cancer-free controls, see Table 1. By matching design, BC patients and controls were identical with regard to age, region of residence, and education as well as gender (they were all women).

**Table 1:** Baseline characteristics of BC patients and cancer-free controls

	BC patients				Cancer-free controls
	Stage ≤ III (diagnosed without distant metastasis)^a^	Stage = IV (diagnosed with distant metastasis)	Stage unknown	Total	
	*N* = 12,715	*N* = 997	*N* = 2793	*N* = 16,505	*N* = 165,042
Age group					
< 45	11%	6%	7%	10%	10%
45–54	21%	12%	16%	20%	20%
55–64	31%	25%	23%	29%	29%
65–74	21%	27%	19%	21%	21%
75+	15%	29%	35%	19%	19%
Region of residence					
Northern Jutland	10%	10%	10%	10%	10%
Central Jutland	21%	22%	19%	20%	20%
Southern Denmark	23%	19%	26%	23%	23%
Capital	30%	29%	31%	30%	30%
Zealand	16%	20%	15%	16%	16%
Education					
Low	38%	49%	37%	38%	38%
Medium	39%	30%	32%	37%	37%
High	19%	11%	14%	18%	18%
Unknown	4%	10%	17%	6%	6%
Cancer treatment started within 9 months from BC diagnosis					
Surgery	96%	63%	81%	91%	N.a.
Chemotherapy	35%	37%	22%	33%	N.a.
Radiation therapy	62%	28%	34%	55%	N.a.
Antibody therapy	4%	7%	2%	4%	N.a.
Hormonal therapy	41%	38%	26%	39%	N.a.

^a^Of the 11,048 BC patients diagnosed in stage I-III in 2004–2007, 31% were diagnosed in stage I, 51% were diagnosed in stage II, and 18% were diagnosed in stage III. Available data did not allow classification of BC patients diagnosed in 2003 according to stage I, II and III

Of the 16,505 BC patients, 12,715 (77%) were diagnosed with early stage BC (i.e. stage I-III without distant metastasis) and 997 (6%) were diagnosed in stage IV (i.e. with distant metastasis), see Table 1. For the remaining 2793 BC patients (17%), cancer disease stage at the time of diagnosis was unknown. Almost all BC patients received surgery (91%), 55% received radiation therapy, 39% received hormonal therapy, 33% received chemotherapy, and 4% received antibody therapy initiated within 9 months of BC diagnosis. A larger proportion of BC patients diagnosed in stage I-III received surgery, radiation therapy and hormonal therapy compared to BC patients diagnosed in stage IV.

In total, 156 per 1000 BC patients (15.6%) were registered with at least one CVD diagnosis in hospital records up to 5 years before the index date (BC diagnosis) compared to 151 per 1000 cancer-free controls (15.1%), see Table 2. The most common type of CVD diagnosis was hypertension (7.3% of BC patients compared to 7.0% of controls) followed by ischemic heart disease/acute coronary syndrome (4.5% of BC patient compared to 4.7% of controls) and cerebrovascular disease (3.7% of BC patients compared to 3.6% of controls). The prevalence rates of CVD were not significantly different between BC patients and controls with the exception of atrial fibrillation (*p* = 0.0019), which was higher in BC patients (3.2% compared to 2.6% in controls).

**Table 2:** Number of persons with CVD diagnosis before the index date per 1000 persons

	BC patients	Cancer-free controls	Difference	*P* value
**CVD**	**156.3**	**150.7**	**5.6**	**0.0546**
Hypertension	73.4	69.6	3.8	0.0687
Ischemic heart disease/ACS	44.9	47.3	−2.4	0.1659
Cerebrovascular disease	36.5	36.4	0.1	0.9409
Atrial fibrillation	30.2	26.1	4.1	0.0019
Heart failure	21.4	20.1	1.4	0.2251
Heart valve disease	9.0	9.1	0.0	0.9745
Thrombophlebitis/thrombosis	8.5	7.6	0.9	0.2017
Other cardiac arrhythmias	8.2	7.3	0.9	0.2189
Peripheral vascular disease	6.7	7.7	−1.1	0.1272
Pulmonary heart disease	4.7	4.0	0.7	0.1907

In total, 447 per 1000 BC patients (44.7%) had a history of CVD drug prescriptions (i.e. drugs related to CVD disease management) when diagnosed with BC compared to 445 per 1000 cancer-free controls (44.5%), see Additional file 1. Diuretics were the most common type of drug used by both BC patients and controls (20%) followed by agents acting on the renin-angiotensin system (14%) and beta blocking agents (11%). There were no significant differences between BC patients and controls with regards to these types of CVD drug prescriptions. However, more controls than BC patients had been treated with antithrombotic agents (11% versus 10%, *p* < 0.01) and lipid modifying agents (7% versus 6%, *p* < 0.001), see Supplementary Table 1.

**Fig. 3:**
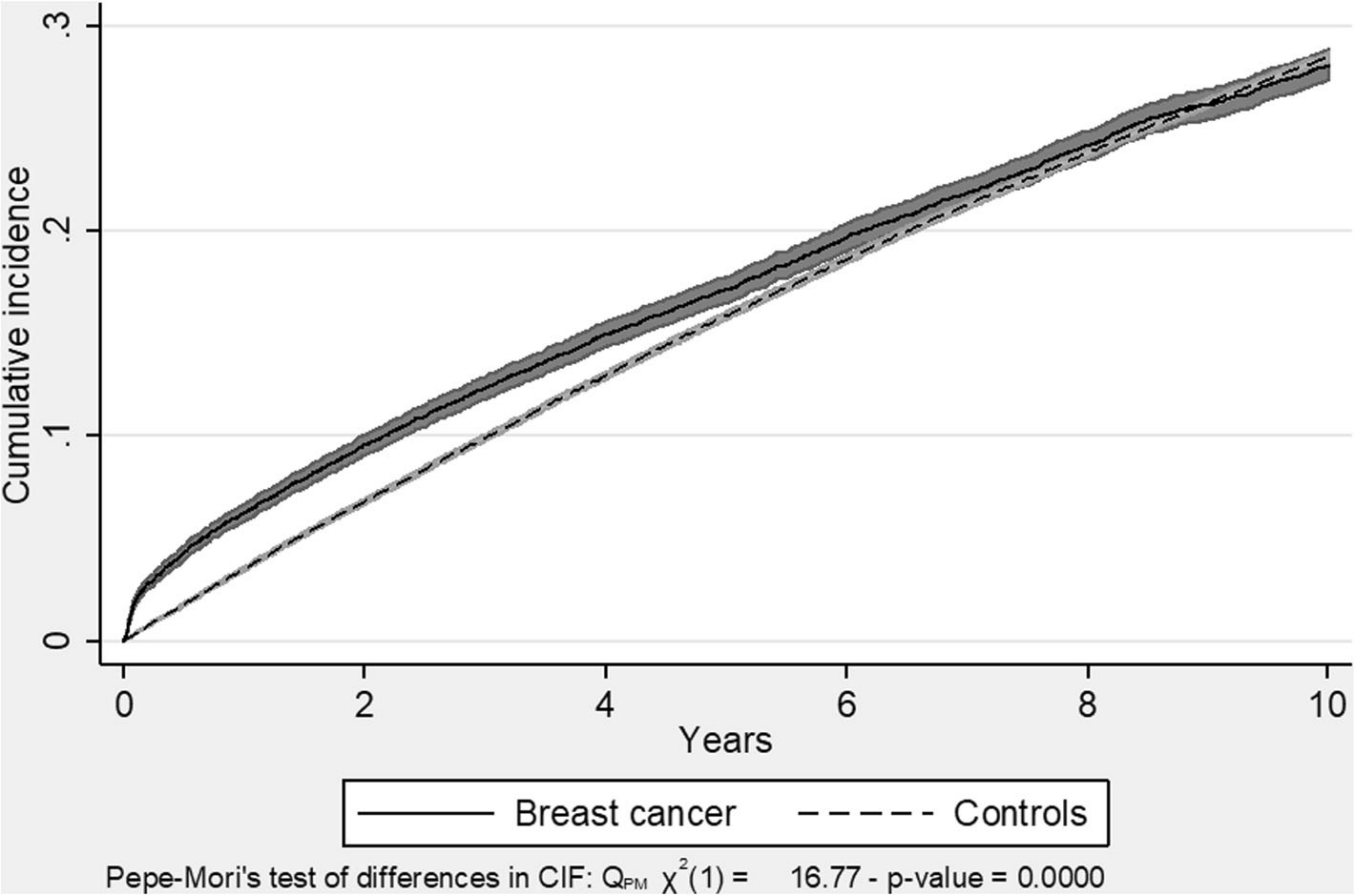
Cumulative incidence of all CVD diagnoses combined, proportion with at least one CVD diagnosis

Up to approximately 6 years after the index date, the cumulative incidence of all CVD diagnoses combined was higher in BC patients than in cancer-free controls taking account of the presence of competing risk of death, see Fig. 3. Hereafter, the difference in cumulative CVD incidence between BC patients and controls was no longer statistically significant. After 10 years, 28% of both BC patients and controls (without any CVD diagnosis in NPR up to 5 years before the index date) had at least one CVD diagnosis in NPR. The most common types of CVD diagnosis were hypertension with a cumulative incidence of 15% after 10 years followed by ischemic heart disease/acute coronary syndrome, cerebrovascular disease and atrial fibrillation (all with a cumulative incidence of 5% after 10 years).

**Fig. 4:**
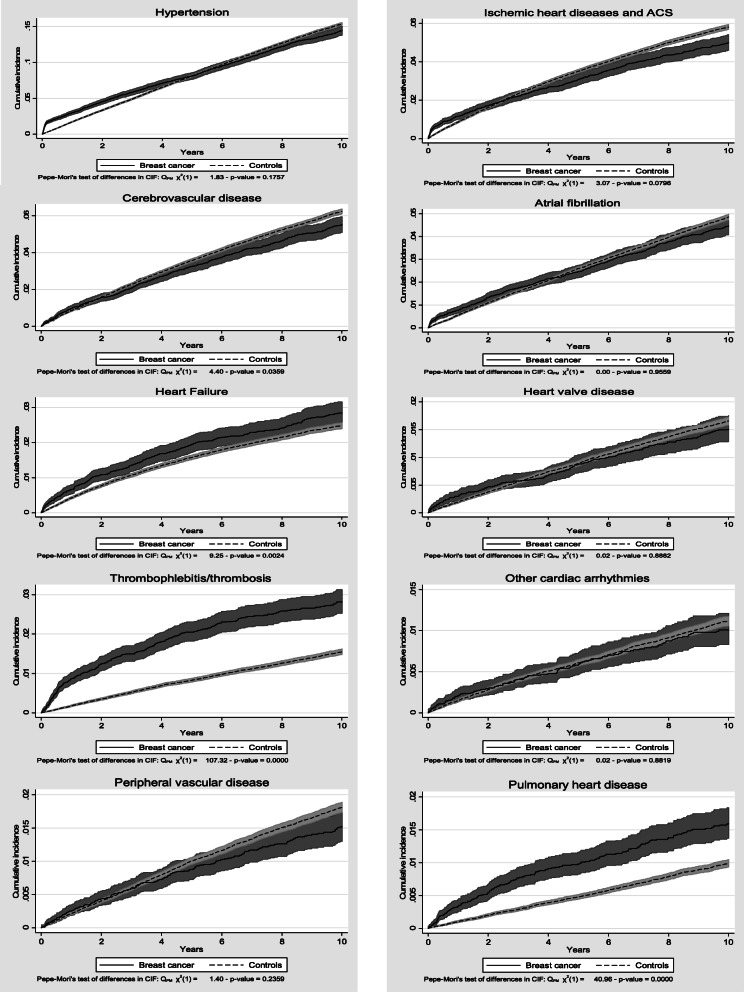
Cumulative incidence of specific types of CVD diagnoses, proportion with CVD diagnosis

During the first 2 years after the index date, the cumulative incidence of most types of CVD was statistically significantly higher in BC patients than controls, see Fig. 4. However, only the cumulative incidence of heart failure, thrombophlebitis/thrombosis and pulmonary heart disease including pulmonary embolism remained higher in BC patients during the entire 10-year follow-up period. After 10 years, 2.7% of BC patients compared to 2.5% of controls had developed heart failure, 2.7% of BC patients compared to 1.5% of controls had developed thrombophlebitis/thrombosis, and 1.5% of BC patients compared to 1.0% of controls had developed pulmonary heart disease.

The risk of CVD was significantly elevated in BC patients after chemotherapy (HR > 1), no matter whether CVD was defined according to CVD diagnoses registered in NPR (95% CI: 1.030–1.353), see Table 3 (model 1), or CVD drug prescriptions registered in NPRD (95% CI: 1.226–1.520), see Table 3 (model 2). Furthermore, the risk of CVD was significantly higher after radiation therapy (HR > 1), when CVD was defined according to CVD drug prescriptions in NPDR (95% CI: 1.023–1.251), but not CVD diagnoses in NPR (95% CI: 0.887–1.117). There were no statistically significant results regarding the risk of CVD after hormonal or antibody therapy.

**Table 3:** Hazard ratio (HR) of CVD in BC patients after different types of cancer treatment

	Model 1: CVD diagnosis in NPR [95% CI]	Model 2: CVD drug prescriptions in NPDR [95% CI]
Chemotherapy	1.180^*^	1.365^***^
	[1.030;1.353]	[1.226;1.520]
Radiation therapy	0.996	1.131^*^
	[0.887;1.117]	[1.023;1.251]
Antibody therapy	1.029	1.032
	[0.759;1.394]	[0.827;1.290]
Hormonal therapy	0.834	0.983
	[0.686;1.014]	[0.832;1.162]
No surgery	1.112	1.091
	[0.951;1.300]	[0.914;1.301]
Distant metastasis when diagnosed with BC	0.978	1.329^*^
	[0.760;1.257]	[1.065;1.659]
Number of observations, N	13.783	8.819

95% confidence intervals in brackets. The following covariates were included in the models: Age, region of residence, education, cohort (year of diagnosis) and comorbidity at index date (CCI, diabetes or CVD (CVD at index date was defined by either CVD drug prescriptions in NPDR (model 1) or CVD diagnoses in NPR (model 2)). Comorbidity at index date was associated with an increased risk of CVD during the follow-up period no matter whether comorbidity was measured by CCI, diabetes or CVD

^*^p < 0.05, ^**^ *p* < 0.01, ^***^ *p* < 0.001

**Fig. 5:**
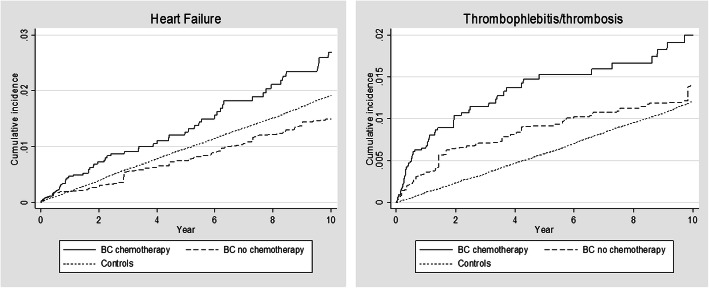
Cumulative incidence of heart failure and thrombophlebitis/thrombosis after chemotherapy, proportion with diagnosis

When investigating the risk of different types of CVD after chemotherapy, we found that the risk of heart failure and thrombophlebitis/thrombosis was significantly elevated in BC patients who received chemotherapy compared to BC patients who did not, see Table 4. Furthermore, we found that the differences in risk of developing heart failure and thrombophlebitis/thrombosis increased during the 10-year follow-up period, see Fig. 5.

**Table 4:** Hazard ratio (HR) of different types of CVD in BC patients after chemotherapy

	Hypertension	Cerebrovascular disease	Ischemic heart disease/ACS^a^	Atrial fibrillation	Heart failure	Thrombophlebitis/thrombosis	Pulmonary heart disease	Peripheral vascular disease	Heart valve disease	Other cardiac arrhythmias
Chemotherapy	0.849	1.012	1.004	1.189	1.738**	1.737**	1.408	0.962	1.014	1.130
Number of observations, N	15,574	16,000	15,874	16,080	16,245	16,387	16,453	16,411	16,388	16,396

The following covariates were included in the model: Age, region of residence, education, cohort (year of diagnosis), distant metastasis when diagnosed with BC and comorbidity at index date (CCI, diabetes or CVD (CVD at index date was defined by CVD drug prescriptions in NPDR

^*^p < 0.05, ^**^ *p* < 0.01, ^***^ *p* < 0.001

^a^ACS = Acute Cornoary Syndrome

## Discussion

We found that 15.6% of BC patients were registered with at least one CVD diagnosis in hospital records up to 5 years before BC diagnosis, and a considerably higher percentage of patients had a history of CVD drug prescriptions (i.e. drugs related to CVD disease management). Overall, BC patients and cancer-free controls were similar with regard to CVD comorbidity at index date. The most common types of CVD diagnoses in both BC patients and controls were hypertension, ischemic heart disease/acute coronary syndrome, and cerebrovascular disease.

After BC diagnosis, the incidence of CVD was significantly higher in BC patients than controls for all CVD diagnoses combined up to approximately 6 years after the index date (BC diagnosis) taking account of the presence of competing risk of death. Higher incidence of CVD in BC patients may be related to cancer treatments such as anthracycline chemotherapy, HER2-targeted agents, aromatase inhibitors, and radiation therapy. It may also be related to lifestyle factors such as obesity and sedentary behaviour if BC patients are more exposed to these risk factors than controls on average.

However, 28% of both BC patients and controls (without any CVD diagnosis in NPR 5 years before the index date) had at least one CVD diagnosis in NPR 10 years after the index date. Only the cumulative incidence of heart failure, thrombophlebitis/thrombosis and pulmonary heart disease including pulmonary embolism remained higher in BC patients than controls during the entire 10-year follow-up period. Furthermore, the risk of heart failure and thrombophlebitis/thrombosis was significantly higher in BC patients who had received chemotherapy compared to BC patients who had not as well as cancer-free controls. Heart failure has been associated with chemotherapy in previous studies [22], and it is well established that cancer patients have increased risk of thrombotic complications including e.g. deep vein thrombosis, pulmonary embolism and arterial thrombosis [23].

Contrary to our results, Abel-Qadir et al. [9] found that the prevalence of different types of CVD was significantly higher in BC patients compared to cancer-free controls. A possible explanation for this discrepancy could be that Abel-Qadir et al. used different algorithms to determine the presence of CVD preceding the index date. Other possible explanations are differences in the definition of the study population and matching criteria, as Abel-Qadir et al. included women diagnosed with early stage BC only, and controls were matched to BC patients according to gender and age, but not education as in our study.

Abel-Qadir et al. [9] found a significantly higher incidence of CVD hospitalizations due to heart failure, cerebrovascular disease and arrhythmias in BC patients compared to cancer-free controls whereas we did not find this for cerebrovascular disease or arrhythmias. In accordance with our results, Strongman et al. [10] found a statistically significantly higher incidence of heart failure and venous thromboembolism in BC patients compared to cancer-free controls, but not significant differences in the incidence of arrhythmia, stroke or peripheral vascular disease. Furthermore, Strongman et al. found that the incidence of coronary artery disease was significantly lower in BC patients compared to controls.

Existing studies have shown an elevated risk of CVD in BC patients after anthracycline-based chemotherapy, radiation therapy and antibody therapy [9, 24–26]. Our study confirms an elevated risk of CVD after chemotherapy. Unfortunately, data available for our study did not allow distinction between different types of chemotherapy. We also saw a tendency towards a higher risk of CVD after radiation and antibody therapy, but these results were in general not statistically significant.

Hormonal agents like tamoxifen approved for BC treatment more than 30 years ago can have both beneficial and detrimental effects on the cardiovascular system [22]. Studies have shown that tamoxifen has a protective effect on lipid metabolism [27–29], but at the same time increases the risk of venous thrombosis and thromboembolism [30]. In our study, we did not find a higher risk of CVD after hormonal therapy, but we saw a higher incidence of thrombotic complications in BC patients compared to cancer-free controls.

The present study has several strengths. Firstly, it is a study at the population level based on a large real-world dataset with no selection bias or other potential issues related to more segmented and selective populations. Secondly, the study population was identified from CAR, which has high completeness and validity due to use of notifications from different data sources and quality control [12]. Thirdly, we used prescription data from NPRD to identify CVD patients as a supplement to data from NPR, as the latter registry does not include patients who require primary care only. NPRD is the national registry of prescription drugs dispensed from community pharmacies and is considered both complete and valid from 1995 and onwards [16]. Fourthly, the study included a cancer-free control group, and the use of exact matching ensured that BC patients and controls were comparable with regard to age, region of residence, and education as well as gender (they were all women). We did not include CCI as a matching criteria to avoid overmatching, and we preferred exact matching to propensity score matching because exact matching ensures that groups are identical with regard to the matching criteria used. Finally, we allowed for the presence of competing risk of death when estimating the cumulative incidence function [20, 31]. Since BC patients had a higher risk of dying during the 10-year follow-up period than controls, ignoring the presence of competing risk could result in substantial bias.

The study also has limitations. Firstly, the study relies on diagnosis and procedure codes in NPR, which may contain errors or be incomplete, e.g. many doctors code the primary diagnosis only, even if the patient has other relevant diseases [32]. Secondly, NPR does not contain information on patients who require primary care only as mentioned above, and it is not straightforward to identify CVD patients based on NPRD data as prescription drugs used for CVD disease management may have other indications. Furthermore, some patients are not diagnosed and consequently not included in NPR or NPRD. Thirdly, drug adherence cannot be ascertained based on NPRD data. If CVD drug adherence is lower in BC patients compared to cancer-free controls, this may be an area of intervention to reduce the risk of serious CVD events in BC patients, but we have no reason to suspect this. Fourthly, as our study population is relatively homogeneous with regard to race, our results may be less generalizable to more diverse patient populations, especially given higher rates of CVD risk factors in some racial minorities. Still, the patient population in Denmark is comparable to most Northern European countries*.* Finally, there is a risk of confounding due to the observational nature of the study. The risk of confounding related to observable baseline characteristics was minimised by exact matching and a multivariable regression design, but BC patients and controls may differ on non-observable lifestyle factors, that influence the risk of CVD. However, we expect lifestyle factors to be similar in BC patients and controls because education was included as a matching criteria in our study and is considered to be a proxy for lifestyle. Similar comorbidity at index date also indicates a comparable lifestyle between BC patients and controls.

## Conclusions

In conclusion, we found that 15.6% of BC patients were registered with at least one CVD diagnosis in hospital records when diagnosed with BC. Overall, BC patients and controls were similar with regard to CVD comorbidity before BC diagnosis. After BC diagnosis, the incidence of all CVD diagnoses combined was significantly higher in BC patients than controls up to approximately 6 years after the index date. The incidence of heart failure, thrombophlebitis/thrombosis and pulmonary heart disease including pulmonary embolism remained higher in BC patients than controls during the entire 10-year follow-up period. Our study supports the need for strategies and novel approaches to minimise and manage the risk of CVD in BC patients. Special attention should be given to reduce the risk of heart failure, thrombophlebitis/thrombosis and pulmonary heart disease including pulmonary embolism in BC patients in the future.

## Supplementary Information

**Additional file 1: Table S1.** Number of persons with CVD drug prescription before the index date per 1000 persons.

## Data Availability

The data that support the findings of this study are available from the Danish Health Data Authority and Statistics Denmark but restrictions apply to the availability of these data, which were used under license for the current study, and so are not publicly available.

## Abbreviations

ATC: Anatomical Therapeutic Chemical Classification System
BC: Breast cancer
CAR: The Danish Cancer Registry
CCI: Charlson Comorbidity Index
95% CI: 95% confidence interval
CVD: Cardiovascular disease
HR: Hazard ratio
ICD: International Classification of Diseases
NPRD: Danish Prescription Drug Registry
NPR: the Danish National Patient Registry
