# Supplementary material for: Cardiovascular disease in women with breast cancer – a nationwide cohort study
Source: BMC Cancer. 2021 Sep 18;21:1040. doi: 10.1186/s12885-021-08716-5 (PMC8449438; doi:10.1186/s12885-021-08716-5)
Supplement: Supplementary file 1 — Additional file 1: Table S1. Number of persons with CVD drug prescription before the index date per 1000 persons. [file 12885_2021_8716_MOESM1_ESM.pdf]

Supplementary Table 1 Number of persons with CVD drug prescription before the index date per 1,000 persons

	BC patients	Controls	Difference	P-value
**Any CVD drug prescriptions**	**446.7**	**444.9**	**1.7**	**0.6699**
Antithrombotic agents (B01)	100.6	108.7	-8.1**	0.0014
Anti-arytmica (C01B)	2.4	2.5	-0.1	0.7955
Antihypertensives (C02)	4.8	4.5	0.3	0.6316
Diuretics (C03)	199.6	199.2	0.4	0.9046
Peripheral vasodilators (C04)	0.5	0.2	0.2	0.0681
Vasoprotectives (C05)	35.1	34.7	0.4	0.7799
Betablocking agents (C04)	108.2	106.4	1.8	0.4684
Calcium channel blockers (C08)	87.7	88.7	-1.0	0.6524
Renin-angiotensin system (C09)	142.6	143.5	-1.0	0.7349
Lipid modifying agents (C10)	57.9	65.1	-7.1***	0.0004

* $p < 0.05$, ** $p < 0.01$, *** $p < 0.001$
